# Cognitive and cerebral phenotypes of neurocognitive disorders due to alcohol or Alzheimer’s disease

**DOI:** 10.1093/braincomms/fcaf289

**Published:** 2025-08-20

**Authors:** Célia Soussi, Shailendra Segobin, Nicolas Cabé, Alice Laniepce, Laurent Coulbault, Céline Boudehent, Vincent de la Sayette, Gaël Chételat, Anne-Lise Pitel

**Affiliations:** Normandie Univ, UNICAEN, INSERM, U1237, PhIND ‘Physiopathology and Imaging of Neurological Disorders’, NeuroPresage Team, Cyceron, Caen 14000, France; Normandie Univ, UNICAEN, PSL Université, EPHE, INSERM, U1077, CHU de Caen, GIP Cyceron, NIMH, Caen 14000, France; Normandie Univ, UNICAEN, INSERM, U1237, PhIND ‘Physiopathology and Imaging of Neurological Disorders’, NeuroPresage Team, Cyceron, Caen 14000, France; Service d'Addictologie, Centre Hospitalier Universitaire de Caen, Caen 14000, France; Normandie Univ, UNICAEN, INSERM, U1237, PhIND ‘Physiopathology and Imaging of Neurological Disorders’, NeuroPresage Team, Cyceron, Caen 14000, France; Normandie Univ, UNIROUEN, CRFDP (EA 7475), Rouen 76000, France; Normandie Univ, UNICAEN, INSERM, U1237, PhIND ‘Physiopathology and Imaging of Neurological Disorders’, NeuroPresage Team, Cyceron, Caen 14000, France; Service de Biochimie, Centre Hospitalier Universitaire de Caen, Caen 14000, France; Normandie Univ, UNICAEN, INSERM, U1237, PhIND ‘Physiopathology and Imaging of Neurological Disorders’, NeuroPresage Team, Cyceron, Caen 14000, France; Service d'Addictologie, Centre Hospitalier Universitaire de Caen, Caen 14000, France; Normandie Univ, UNICAEN, INSERM, U1237, PhIND ‘Physiopathology and Imaging of Neurological Disorders’, NeuroPresage Team, Cyceron, Caen 14000, France; Service de Neurologie, Centre Hospitalier Universitaire de Caen, Caen 14000, France; Normandie Univ, UNICAEN, INSERM, U1237, PhIND ‘Physiopathology and Imaging of Neurological Disorders’, NeuroPresage Team, Cyceron, Caen 14000, France; Normandie Univ, UNICAEN, INSERM, U1237, PhIND ‘Physiopathology and Imaging of Neurological Disorders’, NeuroPresage Team, Cyceron, Caen 14000, France; Institut Universitaire de France (IUF), Paris 75005, France

**Keywords:** Alzheimer’s disease, alcohol-related disorders, cognition, magnetic resonance imaging (MRI), fluorodeoxyglucose positron emission tomography (FDG PET)

## Abstract

Distinguishing aetiologies of neurocognitive disorder (NCD) between alcohol-induced pathologies (OH) and Alzheimer’s disease poses a major clinical challenge while dual diagnosis may be common. We aimed to define commonalities and specificities of neurocognitive alterations in OH or Alzheimer's Disease, considering the NCD severity (mild/major). In this retrospective cross-sectional study, we included 203 participants: 50 Mild-NCD-OH patients, 18 Major-NCD-OH patients, 30 Mild-NCD-AD patients, 24 Major-NCD-AD patients, as well as 81 healthy controls. Patients were compared on a neuropsychological and multimodal neuroimaging assessment (grey/white matter density and glucose metabolism). Analyses explored commonalities and specificities of each patient group within each NCD severity. All patient groups had episodic memory impairments, medial temporal lobe damage and hypometabolism in thalami and posteromedial cortex. NCD-AD patients had more severe cognitive deficits than NCD-OH patients, and the reverse pattern was observed for brain damage. NCD-OH patients notably showed more severe thalamic and cingulate alterations. NCD-OH patients also presented cerebellar damage not observed in NCD-AD. Volume deficits in the medial temporal lobe and memory deficits were more severe in Mild-NCD-AD than Mild-NCD-OH, but similar in Major-NCD-AD and Major-NCD-OH. Common alterations are observed in NCD-OH and NCD-AD, mainly within the memory circuit. Only cerebellar damage appears to be specific to NCD-OH. The specificity of NCD-AD deficits relies on their severity since they are also present to a lesser extent in NCD-OH, reinforcing how the neurocognitive phenotypes overlap. These results reaffirm the importance of questioning alcohol consumption in NCD-AD patients and considering an Alzheimer's Disease diagnosis in NCD-OH patients.

## Teaser

Soussi *et al*. compared profiles of patients with mild or major neurocognitive disorders due to alcohol or Alzheimer’s disease. Cerebellar damage appears specific to alcohol-related disorders. Otherwise, most of the alterations overlapped (notably in memory-related brain regions). This underscores how one aetiology may mask the other in clinical practice.

## Graphical Abstract

**Graphical Abstract:**
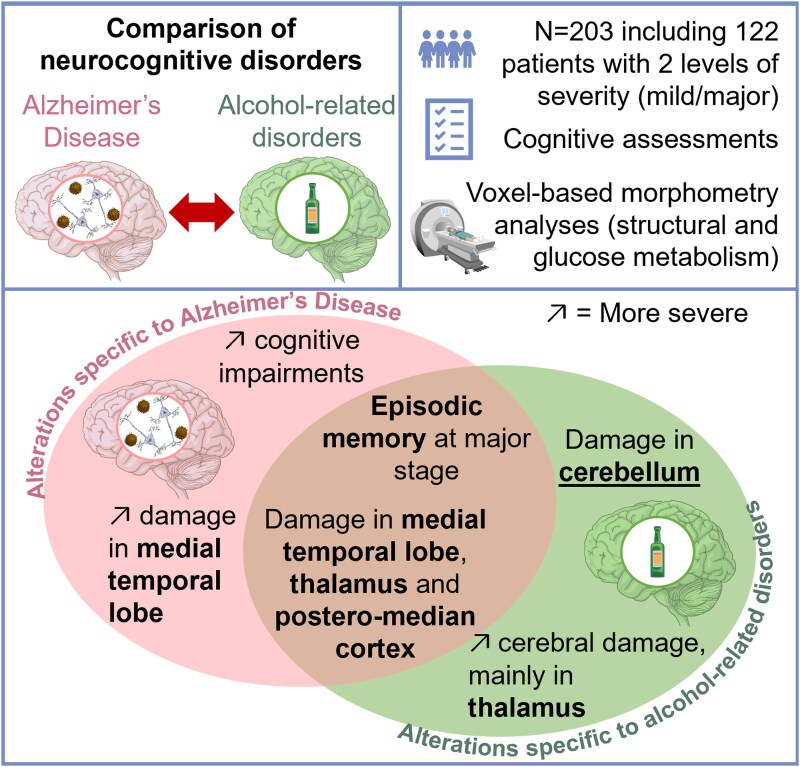

## Introduction

Psychiatrists in Addiction departments struggle when facing an elderly individual with chronic and excessive alcohol consumption who exhibits neurocognitive alterations suggestive of Alzheimer’s disease. Meanwhile, questioning alcohol consumption and evaluating its impact in patients with a primary diagnosis of a neurodegenerative disease such as Alzheimer's Disease remains complex and is not systematically done. These challenging situations underline the need to bridge the gap between psychiatry and neurology to identify the specific aetiology responsible for the patient’s symptoms, which is essential for treatment and clinical orientation. These situations can be encountered for different severities of neurocognitive disorder (NCD) as defined by the DSM-5: either mild, as in alcohol use disorder (Mild-NCD-OH) and amnesic mild cognitive impairment (Mild-NCD-AD), or major in Korsakoff’s Syndrome (Major-NCD-OH) and Alzheimer's Disease at a dementia stage (Major-NCD-AD).^1^

Both aetiologies represent important public health burdens: Alzheimer's Disease is the principal cause of dementia worldwide^2^ and Mild-NCD-OH is one of the most prevalent mental diseases.^3^ Dual diagnosis can also be frequent: while a late onset of alcohol abuse can be a symptom of dementia,^4^ excessive alcohol consumption is a risk-factor for dementia.^2^ Mild-NCD-OH is amongst the most significant modifiable risk-factor for all types dementia and is the main modifiable risk-factor for early onset dementia.^5^ Regarding the onset of Alzheimer's Disease more specifically, main modifiable risk-factors are both Mild- and Major-NCD-OH.^5^ In addition, both preclinical^6^ and clinical studies^7^ suggest that excessive alcohol consumption may accentuate Alzheimer's Disease pathology. A study investigating biological biomarkers of Alzheimer's Disease in old patients with alcohol-related NCD underscore the lack of reliable Alzheimer's Disease biomarkers in the context of heavy and chronic alcohol consumption.^8^ Clinicians may face significant challenges in managing such cases, highlighting the need for neurocognitive biomarkers. Improving care of patients in whom both Alzheimer's Disease and alcohol-related pathologies are suspected requires more evidence on cognitive deficits and brain alterations specific to Alzheimer's Disease and alcohol, considering severity of neurocognitive symptoms.

Direct comparison of cognitive or cerebral profiles between Mild-NCD-OH and Mild-NCD-AD has not yet been reported. Regarding Major-NCD, neuropsychological profiles of Major-NCD-OH and Major-NCD-AD patients have been compared.^9^ Major-NCD-OH and Major-NCD-AD being respectively described as diencephalic or hippocampal amnesia, these previous studies mainly specified the precise pattern of memory performance in each group.^10-12^ For example, episodic deficits are associated with retrieval in Major-NCD-OH but storage in Major-NCD-AD.^12^ Concerning neuroimaging investigations, only two investigations compared profiles of abnormalities in similarly cognitively impaired Major-NCD-OH and Major-NCD-AD patients.^13,14^ These two studies focused on the structural integrity of regions involved in episodic memory.^13,14^ They have highlighted similar volume deficits in Major-NCD-OH and Major-NCD-AD patients in the hippocampi,^13,14^ cingulate cortex^13^ and, anterior thalamic nuclei.^13^ Patients with Major-NCD-AD had lower volume in the temporal cortex than Major-NCD-OH patients,^14^ and the latter had lower volume in the medial thalamic nuclei and mammillothalamic tract than Major-NCD-AD patients.^13^ However, other cerebral substrates involved in memory (e.g. parietal cortices)^15^ were not investigated, as well as brain regions that could be related to impairments in other cognitive functions. In addition, results for other imaging modalities are lacking (e.g. functional modalities or white matter integrity measures).

Our aim is to highlight commonalities and specificities in the patterns of NCD due to alcohol or to Alzheimer's Disease, using a multimodal whole-brain imaging approach that includes both structural and metabolic examinations, combined with a neuropsychological evaluation. It is the first study to directly compare these aetiologies considering two levels of NCD severity (mild/major). This cognitive and cerebral phenotyping will enhance our understanding of potential common pathophysiological mechanisms and will favour better treatment of patients with NCD-OH and/or NCD-AD in clinical settings.

## Materials and methods

### Participants

In this comparative, retrospective cross-sectional study, we used baseline data from two observational and longitudinal studies conducted in the same neuroimaging centre (Cyceron, Caen, France), a facility of France Life Imaging network (grant ANR-11-INBS-0006). Studies were conducted on the same MRI and PET cameras using the same imaging protocol, and a partially shared neuropsychological evaluation. The IMAP study (registered with http://clinicaltrials.gov, number NCT01638949) aimed at investigating the relevance of different brain measures in the prediction of Alzheimer's Disease progression of cognitively healthy adults (HC) and patients at different stages of Alzheimer's Disease (inclusion and data collection between January 2008 and October 2016). ALCOBRAIN/ALCOSLEEP study (registered with http://clinicaltrials.gov, number NCT01455207) aimed at screening for cognitive impairments and brain damage in NCD-OH patients (inclusion and data collection between October 2012 and July 2019). All participants were at least 18 years old and were native French speakers. Participants had no history of cerebrovascular disease, psychiatric disorders, head trauma, drug abuse (except tobacco for all participants and alcohol for NCD-OH patients), neurologic disease (other than KS for Major-NCD-OH patients or Alzheimer's Disease for NCD-AD groups) or other major diseases such as cancer. No participant was under psychotropic medication with the exception of four KS patients who had been on stable medication for several years, with no recent changes. Both the IMAP and ALCOBRAIN/ALCOSLEEP studies were approved by the Comité de Protection des Personnes Nord-Ouest III in France. All participants (or their caregivers when necessary) provided their written informed consent for inclusion in either protocol. These studies were carried out in line with the Declaration of Helsinki (1964).

HC participants had to be cognitively unimpaired and to have performance in the normal range in all neuropsychological tests. NCD-AD patients were recruited in local memory clinics and met DSM-5 criteria for mild or major NCD as well as internationally agreed criteria for probable Alzheimer's Disease. Mild-NCD-AD patients were diagnosed with aMCI^16^ and Major-NCD-AD patients with dAD.^17^ Patients with severe form of dAD were not included to avoid difficulties in carrying out the protocol. Mild-NCD-OH patients were recruited as inpatients in the Addiction department of Caen University Hospital at the end of their withdrawal period [i.e. patients had been free of benzodiazepine withdrawal treatment and symptoms for at least 48 h, corresponding to a mean of 11 ± 4.67 days of abstinence (min = 8; max = 24)] and met the DSM-IV criteria for alcohol-dependence^18^ or DSM-5 criteria for severe AUD.^1^ Major-NCD-OH patients were either recruited as inpatients or in a nursing home, and were diagnosed with KS as defined by the DSM-5 criteria for alcohol-induced major NCD, amnestic-confabulatory type, persistent^1^ or by the DSM-IV criteria for amnesia due to substance abuse.^18^ Part of the latter diagnosis involved patients showing amnesia on repeated neuropsychological assessments. All patients were diagnosed by experienced clinicians after careful investigation. Our different groups have already been included in other studies (e.g. for NCD-OH groups^13,19,20^ and for NCD-AD and HC groups^21,22^).

We only included participants who had performed an MRI examination with satisfactory quality control (e.g. not too many movements during acquisition). Amyloid status of IMAP participants was assessed based on their 18F-AV45 PET (fluorine-18 florbetapir positron emission tomography) cortical SUVr (standardized uptake value ratio) transformed to Centiloids^23,24^ (see Supplementary Method for details). We included Mild- and Major-NCD-AD patients with a positive amyloid status and HC with a negative amyloid status, using a cut-off of 12 Centiloid.^25^

Even if we are using baseline data only, the longitudinal follow-up conducted in IMAP allowed to reassess the Alzheimer's Disease diagnosis up to 15 years after inclusion. A diagnostic committee composed of a multidisciplinary team of experts reassessed Alzheimer's Disease diagnosis, when possible, based on information from the longitudinal study and consultation with clinicians involved in patient care after study completion. Clinical information about NCD-OH patients were also consulted in September 2024 (5–12 years after inclusion) to assess whether any neurodegenerative disease was suspected at distance from inclusion. One Major-NCD-OH patient later received a diagnosis of Alzheimer's Disease and was excluded from the present study.

### Data collection and measurements

#### Neuropsychological examination

Episodic memory was assessed with the delayed free recall of the Free and Cued Selective Reminding Test (FCSRT, French version^26^). An executive functioning score was computed as the sum of the differences in seconds (i) between parts B and A of the trail making test (TMT)^27^ and (ii) between interference and denomination parts of the Stroop test.^28^ Working memory was assessed with the sum of the forward and backward spans from the WAIS-III.^29^ A processing speed score was computed in seconds as the sum of realization time of (i) part A of the TMT^27^ and (ii) denomination part of the Stroop test.^28^ Visuo-construction abilities were measured with the score at the copy (/36) of the Rey–Osterrieth complex figure.^30^

#### Neuroimaging examination

Imaging data were pre-processed and analysed using SPM12 (https://www.fil.ion.ucl.ac.uk/spm/software/spm12/).

#### MRI images pre-processing and analyses

High-resolution T1-weighted anatomical images were obtained with a 3D fast-field echo sequence on a Philips Achieva 3T scanner with the following parameters: 180 sagittal slices; thickness = 1 mm, repetition time (TR) = 20 ms, echo time (TE) = 4.6 ms, flip angle = 10°, field of view (FOV) = 256 × 256 mm^2^. T1-weighted images were segmented into grey matter (GM), white matter (WM) and cerebrospinal fluid. A study-specific template was first created using DARTEL^31^ for both GM and WM. Participants’ images were subsequently projected into Montreal Neurological Institute (MNI) space and modulated to preserve their volumes. Unmodulated images from HC were averaged and threshold at 0.5 to create GM and WM binary masks for statistical analyses. For each participant, modulated images smoothed with an 8-mm^3^ Gaussian kernel were used.

#### PET images pre-processing and analyses

[18F] fluorodeoxyglucose ([18F] FDG) PET images were acquired on a Discovery RX VCT 64 PET-CT scanner with the following parameters: 47 planes; resolution = 3.76 × 3.76 × 4.9 mm^3^; axial field = 157 mm. FDG-PET images were corrected for partial volumes effects (PVE) with a Müller-Gartner method between GM and CSF and between GM and WM. PVE-corrected images were co-registered to participants’ anatomic MRI and normalized to the MNI template.^32^ Semi-quantitative intensity normalization was performed to avoid inter-individual variations in metabolism using cerebellar lobules III, IX and X as reference region, as they are known to be unimpaired in all pathologies under this study.^33,34^ Finally, all images were smoothed with a 10-mm^3^ Gaussian kernel. The GM mask was applied to PET images during statistical analyses.

### Statistics

#### Demographics and cognition

The distribution of all variables was not perfectly normal according to Shapiro–Wilk, but close enough (Skewness > 2 Kurtosis > 7) to allow parametric analysis. We evaluated between groups differences in demographics with χ² and ANOVAs. For each neuropsychological score, an ANCOVA was carried out to evaluate the group effect controlling for sex, age and education. Since our groups differed in terms of demographics (Table 1), we checked the proportion of variance explained by age, sex and education. For all cognitive function, the proportion of the variance is always mainly explained by the group (see Supplementary Table 1). All *post hoc* comparisons were conducted using Tukey’s HSD method to account for different sample sizes. Some patients were unable to complete certain neuropsychological tests. Such missing data were replaced by the worst score of the corresponding patient group. The purpose of this procedure was to avoid the exclusion of the most severely impaired patients from analyses. Randomly missing data were replaced by the mean score of the group. Younger participants from the HC group (20–39 years old, *N* = 34) were not assessed with the FCSRT and the Rey–Osterrieth complex figure during their protocol completion, but with other tests more relevant for this age range. We did not replace these missing data by the mean of the group since performance of older participants would not accurately fit their expected performance. Thus, HC participants with missing data were excluded from episodic memory and visuo-construction analyses. Sample sizes are specified in Table 2. Demographic and neuropsychological statistical analyses were carried out using R Statistical Software (v4.2.3). The following R packages were used in the cognitive analysis: car (version 3.1.2) for Type III ANOVA using the Anova() function; multcomp (version 1.4.25) for *post hoc* Tukey’s HSD tests via glht(); effectsize (version 0.8.6) to compute effect sizes with eta_squared(), in addition to core R functions such as lm(), confint(), and standard plotting tools. Code used is provided in Supplementary Method. Significant results are reported at a 5% alpha-level (*P* < 0.05).

**Table 1:** Demographics and global cognition variables

	Mild-NCD		Major-NCD				
	OH	AD	OH	AD	HC	Statistics	*Post hoc* comparisons
Demographics							
*N*	50	30	18	24	81		
Sex						*X^2^* = 18.31; *P* = 0.001; *V* = 0.29	
Male	44 (88%)	20 (66%)	8 (44%)	16 (66%)	45 (55%)		
Female	6 (12%)	10 (34%)	10 (56%)	8 (33%)	36 (45%)		
Age (years)	46.89 (8.90) [26–66]	73.76 (6.64) [60–85]	54.83 (4.80) [44–62]	67.78 (9.89) [54–84]	47.30 (19.8) [20–81]	*F*(4,198) = 28.37; *P* < 0.001; *η^2^* = 0.36	(HC = NCD-OH) < (NCD-AD)
Education (years)	11.86 (2.10) [9–17]	11.66 (4.17) [6–20]	10.06 (2.26) [6–15]	11.17 (3.40) [6–20]	13.48 (3.19) [7–20]	*F*(4,198) = 6.70; *P* < 0.00; *η^2^* = 0.12	(NCD-OH = NCD-AD) < HC
Global cognition							
*N*	27	30	18	23	81		
MMSE^a^	27.18 (2.70) [20–30]	26.83 (1.82) [22–30]	22.63 (3.68) [12–27]	20.74 (4.93) [12–29]	29.10 (0.99) [26–30]	*F*(4,174) = 60.60; *P* < 0.001; *η^2^* = 0.58	(Major-NCD) < (Mild-NCD) < HC

Data are *N* (%) or mean (SD) [range]. Groups were compared with χ² or ANOVAs. *Post hoc* test: Tukey’s HSD.

Mild-NCD-OH, patients with alcohol use disorder; Mild-NCD-AD, patients with amnesic-type mild cognitive impairment; Major-NCD-OH, patients with Korsakoff’s Syndrome; Major-NCD-AD, patients with Alzheimer’s disease at a dementia stage; MMSE, Mini-Mental State Examination; NA, not applicable.

^a^MMSE data were missing for 23 Mild-NCD-OH patients and 1 Major-NCD-AD patient.

**Table 2:** Neuropsychological scores

	Mild-NCD		Major-NCD				
	OH *n* = 50	AD *n* = 30	OH *n* = 19	AD *n* = 24	HC ^a^*n* = 80 ^b^*n* = 46	Statistics	*Post hoc* comparisons
Episodic memory^c^ (max = 16)	10.42 (3.38) [0–14]	4.50 (3.14) [0–10]	2.78 (3.02) [0–11]	2.04 (2.35) [0–8]	12.91^b^ (2.11) [9–16]	*F*(4,161) = 77.58; *P* < 0.00; *η^2^* = 0.70	(Major-NCD) < Mild-NCD-AD < Mild-NCD-OH < HC
Executive functions^d^ (s)	177.64 (193.49) [33–1039]	197.67 (115.37) [68–700]	307.11 (206.43) [89–701]	875.82 (491.65) [55–1405]	80.47^a^ (37.32) [24–203]	*F*(4,195) = 57.66; *P* < 0.001; *η^2^* = 0.58	Major-NCD-AD < (HC = Mild-NCD) Major-NCD-OH < HC Major-NCD-AD < Major-NCD-OH
Working memory^e^	10.10 (2.28) [5–15]	9.83 (1.72) [6–13]	9.22 (1.70) [7–12]	8.46 (1.96) [5–13]	11.27^a^ (2.21) [7–17]	*F*(4,195) = 4.35; *P* = 0.002; *η^2^* = 0.18	HC = Mild-NCD = Major-NCD-OH Mild-NCD = Major-NCD Major-NCD-AD < HC
Processing speed^f^ (s)	121.22 (40.76) [68–274]	128.84 (22.05) [84–165]	174.33 (74.62) [100–350]	222.70 (84.89) [99–481]	91.83^a^ (18.08) [53–142]	*F*(4,195) = 32.90; *P* < 0.001; *η^2^* = 0.49	Major-NCD-AD < Major-NCD-OH < (HC = Mild-NCD)
Visuo-construction^g^ (max = 36)	33.9 (3.02) [26–36]	33.22 (3.62) [20–36]	30.03 (7.14) [14–36]	22.94 (10.10) [6–36]	34.62^b^ (2.07) [27–36]	*F*(4,161) = 23.93; *P* < 0.001; *η^2^* = 0.38	Major-NCD-AD < (HC = Mild-NCD) Major-NCD-AD < (HC = Mild-NCD-AD = Major-NCD-OH) Major-NCD-OH < Mild-NCD-OH

Data are mean (SD) [range]. Groups were compared with ANCOVAs controlling for age, sex and education. *Post hoc*: Tukey HSD. Data missing at random was replaced by the mean of the group, and data missing due to inability to complete the test was replaced by the worst score of the group. A number of patients concerned by this procedure are specified by test and by group.

Mild-NCD-OH, patients with alcohol use disorder; Mild-NCD-AD, patients with amnesic-type mild cognitive impairment; Major-NCD-OH, patients with Korsakoff’s Syndrome; Major-NCD-AD, patients with dementia stage Alzheimer’s disease.

^a,b^Sample size for the HC group is specified for each cognitive score.

^c^One missing data in the Mild-NCD-OH group, one in the Mild-NCD-AD group and seven in the Major-NCD-AD group were replaced.

^d^Four missing data in the Mild-NCD-OH group, 1 in the Mild-NCD-AD group, 2 in the Major-NCD-AD group and 16 missing data in the Major-NCD-AD group were replaced.

^e^One missing data in the Major-NCD-AD group was replaced.

^f^Two missing data in the Mild-NCD-OH group, one in the Mild-NCD-AD group and two in the Major-NCD-AD group were replaced.

^g^Two missing data in the Mild-NCD-OH group and one in the Major-NCD-AD group were replaced.

#### Imaging data

Voxel-wised analyses were carried out using a full factorial model with the group as a factor and total intracranial volume (TIV), sex, age and education as covariates. Same analyses were run without TIV as covariate for PET modality. The cluster size threshold was set at 200 mm^3^ (*k* = 60 for MRI and *k* = 25 for PET) with an uncorrected *P* < 0.001 threshold. We did not correct our results for multiple comparisons to avoid over-correction and missing expected and clinically relevant results.^35^ To compare brain abnormalities in NCD-OH and NCD-AD, we assessed (i) common patterns of alterations between groups of patients when compared to HC participants using conjunction analyses and (ii) direct differences between patient groups. Comparisons were realized with regard to NCD severity: between Mild-NCD (Mild-NCD-OH versus Mild-NCD-AD patients) and then Major-NCD (Major-NCD-OH versus Major-NCD-AD patients). Conjunction analyses used patient < HC contrasts and identified voxels for which the NCD-OH and NCD-AD groups were significantly altered. To check for the proportion of variance explained by demographics variable, similar models than for cognition were run on extracted values from significant clusters, for each contrast of each modality. The proportion of the variance was almost systematically mainly explained by the group for imaging also (see Supplementary Table 2).

## Results

Fifty Mild-NCD-OH patients (age = 46.89 ± 8.90, 88% male) and 18 Major-NCD-OH patients (age = 54.83 ± 4.80, 44% male) from the ALCOBRAIN/ALCOSLEEP study as well as 30 Mild-NCD-AD patients (age = 73.76 ± 6.64, 66% male), 24 Major-NCD-AD patients (age = 67.78 ± 9.89, 66% male) and 81 HC participants (age = 47.30 ± 19.80, 55% male) from the IMAP study were included in our analyses. Participants were not matched for age, sex and education. Frequency of male was higher amongst Mild-NCD-OH patients. NCD-AD patients were older, and HC participants were more educated (Table 1). We used the Mini-Mental State Examination^36^ to measure global cognitive efficiency. On this test, all patients showed impairments compared to HC and were matched consistently with the NCD severity.

### Neuropsychological examination

Regarding neuropsychological examination (Table 2), Mild-NCD patients differed from HC participants in episodic memory only (Mild-NCD-OH: *t* = 4.379, 95% Confidence Interval (CI) [1.066; 4.659], *P* < 0.001; Mild-NCD-AD: *t* = 10.261, 95% CI [5.136; 8.895], *P* < 0.001). Mild-NCD-AD patients had significantly worse episodic memory performance than Mild-NCD-OH patients (*t* = −4.847, 95% CI [−6.507; −1.798], *P* < 0.001) while their performance was similar in all other investigated functions. Both Major-NCD-OH and Major-NCD-AD patients had impaired performance compared to HC participants in processing speed (respectively: *t* = −6.062, 95% CI [−102.407; −38.642], *P* < 0.001; *t* = −10.298, 95% CI [−143.713; −83.301], *P* < 0.001), episodic memory (respectively: *t* = 13.060, 95% CI [7.890; 12.096], *P* < 0.001; *t* = 14.133, 95% CI [7.880; 11.685], *P* < 0.001) and executive functioning (respectively *t* = −3.057, 95% CI [−889.229; −600.531], *P* = 0.020; *t* = −14.141, 95% CI [−322.337; −17.616], *P* < 0.001). Only Major-NCD-AD patients were impaired compared to HC on working memory (*t* = 3.756, 95% CI [0.524; 3.355], *P* = 0.002) and visuo-construction (*t* = 8.641, 95% CI [7.477; 14.447], *P* < 0.001). Major-NCD-AD patients had significantly worse performance than Major-NCD-OH patients in executive functioning (*t* = −8.811, 95% CI [−753.715; −396.093], *P* < 0.001), processing speed (*t* = −3.148, 95% CI [−80.400; −5.565], *P* = 0.015) and visuo-construction (*t* = 4.962, 95% CI [3.555; 12.374], *P* < 0.001) but similar performances in episodic memory (*t* = −0.240, 95% CI [−2.617.535; 2.197], *P* = 0.999) and working memory (*t* = 1.143, 95% CI [−1.022; 2.485], *P* = 0.777).

### Neuroimaging examination

For all investigated cerebral imaging modalities, profiles of patients compared to HC participants were consistent with the literature and will not be further discussed herein (see Supplementary Figs 1 and 2).

### Grey matter density

**Figure 1:**
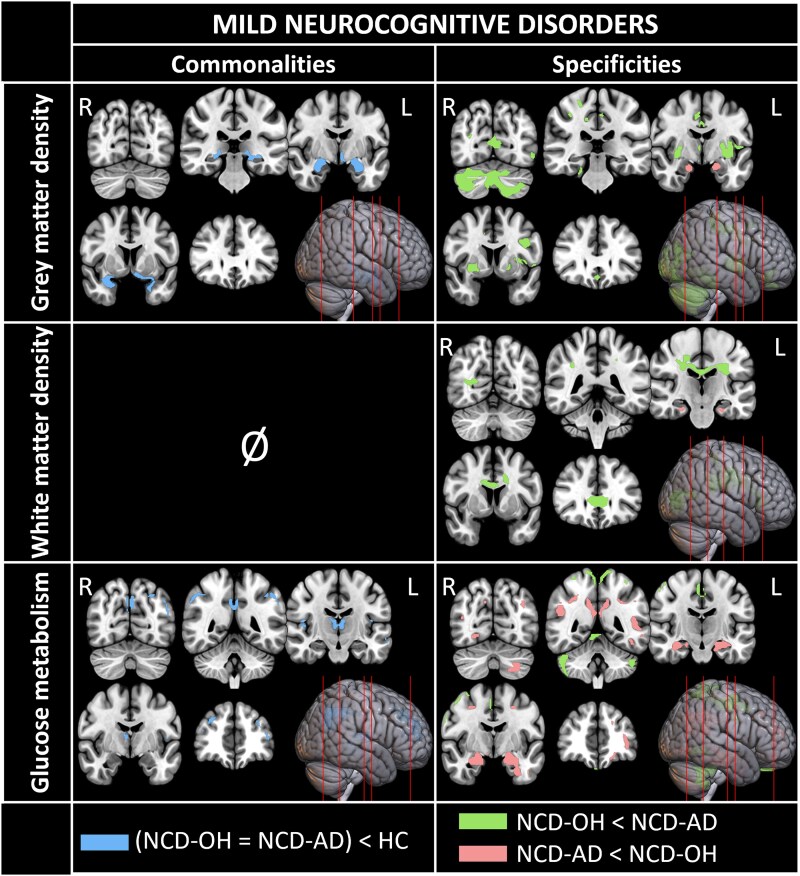
**Neuroimaging results for conjunction analyses and between-group comparisons for mild neurocognitive disorders.** Voxel-wised analyses were carried out using a full factorial model with the group as a factor and total intracranial volume (TIV), sex, age and education as covariates. Same analyses were run without TIV as covariate for PET modality. Significant results (*P* < 0.001, *k* = 200 mm^3^) for each investigated contrast have been projected on five axonal sections and rendered on a lateral view of the brain. Each row corresponds to an imaging modality (from *top* to *bottom*: grey matter density, white matter density, glucose metabolism). *Left* column displays results for the conjunction analysis of both patients’ groups (Mild-NCD-OH *n* = 50; Mild-NCD-AD *n* = 30) compared to controls participants (HC *n* = 80). *Right* column displays results for comparison between patients’ groups, legend in the *bottom* row indicates colour codes for each contrasts investigated (green: Mild-NCD-OH patients < Mild-NCD-AD patients; pink: Mild-NCD-AD patients < Mild-NCD-OH patients). For the glucose metabolism modality, the sample size for the Mild-NCD-OH group was *n* = 42.

Regarding GM density analyses, the conjunction analysis showed that Mild-NCD patients had common damage in the amygdala, medial temporal lobe and superior temporal pole (Fig. 1). Direct patient group comparisons showed that Mild-NCD-OH patients had more severe alterations than Mild-NCD-AD patients in the frontal lobe, middle cingulate cortex, parietal lobe, left inferior and middle temporal gyri, left superior temporal pole, occipital cortices, left insula, thalamus, putamen and cerebellum. Mild-NCD-AD patients had more severe damage than Mild-NCD-OH patients in the amygdala, anterior hippocampi and parahippocampal gyrus.

**Figure 2:**
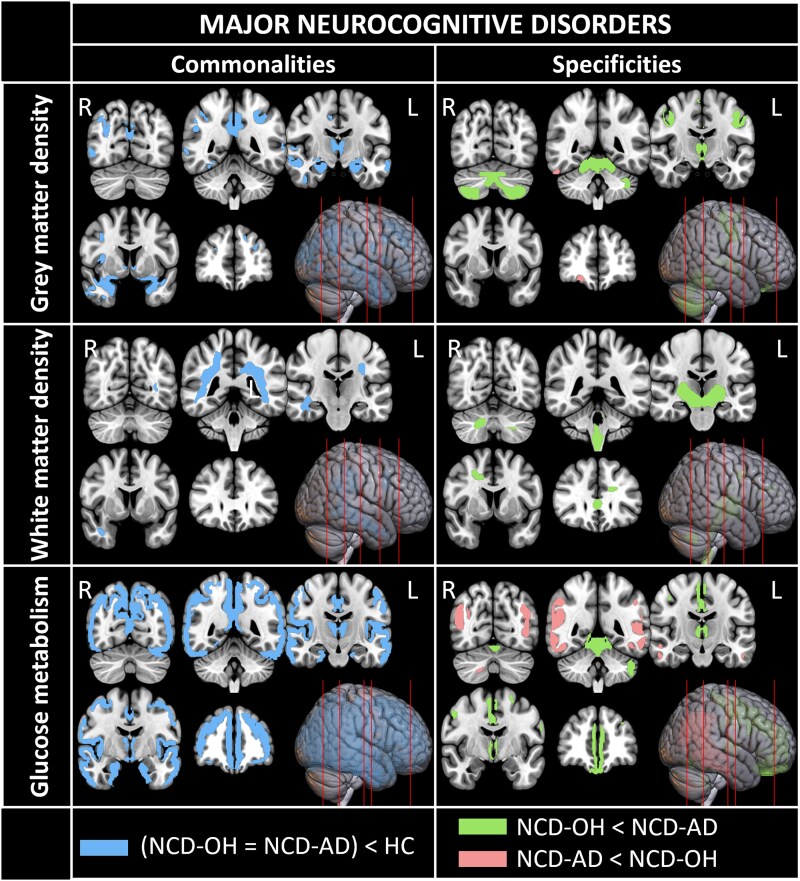
**Neuroimaging results for conjunction analyses and between-group comparisons for major neurocognitive disorders.** Voxel-wised analyses were carried out using a full factorial model with the group as a factor and total intracranial volume (TIV), sex, age and education as covariates. Same analyses were run without TIV as covariate for PET modality. Significant results (*P* < 0.001, *k* = 200 mm^3^) for each investigated contrast have been projected on five axonal sections and rendered on a lateral view of the brain. Each row corresponds to an imaging modality (from *top* to *bottom*: grey matter density, white matter density, glucose metabolism). *Left* column displays results for the conjunction analysis of both patients’ groups (Major-NCD-OH *n* = 18; Major-NCD-AD *n* = 24) compared to controls participants (HC *n* = 80). *Right* column displays results for comparison between patients’ groups, legend in the *bottom* row indicates colour codes for each contrasts investigated (green: Major-NCD-OH patients < Major-NCD-AD patients; pink: Major-NCD-AD patients < Major-NCD-OH patients). For the glucose metabolism modality, the sample size for the Major-NCD-AD group was *n* = 23.

Regarding Major-NCD patients (Fig. 2), the two groups shared widespread cortical damage (frontal, cingulate, parietal, temporal, insula and occipital) as well as volume deficits in the amygdala, thalami, caudate nuclei and left cerebellum. Major-NCD-OH patients had significantly more severe damage to the left inferior frontal gyrus, central regions, left middle cingulate cortices, thalami, mamillary bodies and cerebellum than Major-NCD-AD patients. Conversely, Major-NCD-AD patients had further alterations than Major-NCD-OH patients in small clusters located in the left inferior occipital gyri, and right middle and superior frontal gyri.

### White matter density

No common volume deficit was found for Mild-NCD patients in WM analyses (Fig. 1). Mild-NCD-OH patients presented more severe damage than Mild-NCD-AD patients in the corpus callosum, cingulum and left occipital WM. Only the bilateral ventral part of the cingulum was more severely damaged in Mild-NCD-AD than in Mild-NCD-OH patients.

For Major-NCD patients (Fig. 2), common impairments were mainly located in parietotemporal WM regions: arcuate anterior and posterior segments, inferior longitudinal fasciculus and left cingulum. Major-NCD-OH patients showed significantly more severe damage to the cerebellum WM, brainstem, diencephalon and parieto-frontal WM regions than Major-NCD-AD patients, with lower volume in the fornix, corpus callosum, cingulum, superior cerebellar pedunculus, posterior limb of the internal capsule and corticospinal tracts. Major-NCD-AD patients did not exhibit more severe WM damage than Major-NCD-OH patients.

### Glucose metabolism

Regarding glucose metabolism analyses, Mild-NCD patients shared lower metabolism than HC participants in the left dorsolateral frontal gyri, posteromedial cortex (precuneus, posterior and middle cingulate), angular gyrus and thalami (Fig. 1). The brain metabolism of Mild-NCD-OH patients was lower than that of Mild-NCD-AD patients in the supplementary motor area, middle cingulate cortex, superior parietal gyrus, inferior precuneus and bilateral cerebellum. Lower metabolism was observed in Mild-NCD-AD patients compared to Mild-NCD-OH patients in the right dorsolateral superior frontal gyrus and orbital part of the left middle frontal gyrus, posterior and middle cingulate cortices, posterior precuneus, temporal cortex and medial temporal lobe and left cerebellar lobule VIII.

For Major-NCD patients (Fig. 2), posteromedial cortices were the most significantly impaired regions in both patient groups. Shared hypometabolism was widespread in bilateral cortices (frontal, anterior cingulate, parietal, temporal, insula and occipital areas), as well as in the medial temporal lobe and right superior temporal pole, thalami and caudate. Major-NCD-OH patients had lower metabolism than Major-NCD-AD patients in frontal and cingulate cortices, thalami and cerebellum. Major-NCD-AD patients had lower metabolism than Major-NCD-OH patients in supramarginal and angular gyri, temporal gyri, fusiform gyrus, middle occipital gyrus and right cerebellar lobule VIII. Hypometabolism in the cerebellar lobule VIII was observed in NCD-AD patients when compared to NCD-OH patients. This can be attributed to the hypermetabolism observed in NCD-OH patients when compared to control participants (data not shown), consistently with the cerebellar maladaptive plasticity previously described in this population.

## Discussion

This study is the first to directly compare NCD related to alcohol versus Alzheimer's Disease, at different levels of severity, using both neuropsychological testing and a multimodal imaging. We aimed at identifying commonalities and specificities of each aetiology by comparing neurocognitive phenotypes. While NCD-AD was on the overall associated with more severe cognitive impairments, brain abnormalities were globally more severe and widespread in NCD-OH, especially regarding white matter density. For cognitive functioning, the two Mild-NCD groups differed from each other only on episodic memory performance, whereas episodic memory scores did not differ significantly between Major-NCD-AD and Major-NCD-OH patients. Regions commonly damaged, whatever the aetiology, in both Mild- and Major-NCD, were the medial temporal lobe for grey matter density and the thalamus and posteromedial cortex for glucose metabolism. Cerebellar structural and functional damage seemed specific to NCD-OH patients and relevant to differentiate these pathologies from Alzheimer's Disease.

The structural integrity of the medial temporal lobe and the metabolism of the thalamus and posteromedial cortex were commonly affected in all clinical populations, consistently with independent observations previously reported for each patient group.^15,20^ Present voxel-based results are in agreement with previous volumetric studies, including one with a Major-NCD-OH population patients that strongly overlaps with ours,^13^ which observed similar damage of the hippocampus,^13,14^ anterior thalamic nuclei^13^ and cingulate cortex^13^ in Major-NCD-OH and Major-NCD-AD patients. However, they also found lower volume of the entire thalamus in Major-NCD-OH patients.^13^ Herein, the whole-brain approach used does not allow analysis of nuclei, but similar thalamic damage in Major-NCD-OH and Major-NCD-AD patients may affect the anterior part.^13^ For both Mild- and Major-NCD, the posterior part of the cingulate cortex appears to be functionally damaged to the same extent in NCD-OH and NCD-AD. White matter analysis showed common damage in Major-NCD only, with shared damage of the left cingulum in parietotemporal regions. The hippocampus, anterior thalamic nuclei, cingulate cortex and cingulum are part of the Papez Circuit, known to support memory functions.^15^ Because alterations of the Papez Circuit are present in both NCD-OH and NCD-AD, we can hypothesize that the increased vulnerability of NCD-OH patients to Alzheimer's Disease pathology^5^ may be linked to compromised integrity of this network. Shared neuropathological processes involved in both alcohol-related pathologies and Alzheimer's Disease may target regions of the Papez Circuit. Early disruption of the serotonergic system may increase vulnerability to Alzheimer's Disease in alcohol abuse.^37^ Disruption of serotonin signalling has direct repercussions on both the hippocampus^38^ and the thalamus,^39^ which could make these regions more vulnerable to neurodegenerative processes seen in Alzheimer's Disease. Additionally, serotonergic dysfunction could lead to increased neuroinflammation, which may further impair amyloid clearance and exacerbate the progression of amyloid pathology in these key Papez Circuit regions. More globally, it has been proposed that neuroinflammation found in NCD-OH patients, both directly due to alcohol exposure and indirectly (via thiamine deficiency, hepatic pathologies, traumatic injuries, etc.), could trigger and/or accelerate amyloid pathology.^40^ For example, neuroinflammation impairs amyloid clearance via dysregulated microglial function.^40,41^ Both hippocampus and thalamus having high microglial reactivity, these two hubs of the Papez Circuit are thus particularly vulnerable to neuroinflammation. Alcohol-related serotoninergic dysfunction and neuroinflammation could leave the Papez Circuit more vulnerable when exposed to Alzheimer's Disease pathology, more especially the thalamus and hippocampus that are targeted early on by Alzheimer's Disease pathology.^15,42^

The brain damage specific to NCD-OH when compared to NCD-AD, at the Mild- or Major-stages, included the presence of cerebellar alterations, more severe volume loss and hypometabolism in the anterior/middle cingulate cortex and thalamus, and more widespread white matter abnormalities. Cerebellar damage could help to identify alterations due to alcohol since the cerebellum is generally preserved in Alzheimer's Disease.^34^ We observed both lower cerebellar volume and metabolism in NCD-OH groups when compared to NCD-AD groups. Development of cerebellar abnormalities, notably in NCD-AD patients, could reflect brain damage due to alcohol. However, cerebellar alterations appear characteristic to NCD-OH at the group level, but not at subject level due to the population’s inherent heterogeneity. White matter alterations could also be considered as specific to alcohol-related damage. For instance, distortion of the corpus callosum is explained by direct white matter pathology in NCD-OH patients but by indirect ventricular expansion in NCD-AD patients.^43^ Characteristic alcohol-related alterations of white matter structures (i.e. shrinkage of the fornix and of the corpus callosum)^44^ could help the clinician to identify NCD-OH. However, neither the presence of cerebellar abnormalities nor of white matter alterations is sufficient to exclude an additional Alzheimer's Disease pathology in a context of dual diagnosis.

In terms of AD-specific damage, the volume of the anterior hippocampus, as well as of a small ventral portion of the right cingulum, was lower in Mild-NCD-AD patients than in Mild-NCD-OH patients. Mild-NCD-AD patients also had lower metabolism in the medial temporal lobe and in the posterior precuneus, as well as more severe episodic memory impairments when compared to Mild-NCD-OH patients. Although medial temporal lobe damage^33^ and memory deficits^45^ are repeatedly found in Mild-NCD-OH patients, a repeated imaging examination showing a degradation centred on the MTL could alert the clinician on the potential development of Alzheimer's Disease, alike a neuropsychological evaluation revealing a steeper worsening of episodic memory performance. In the same way, in case of abstinence, or at least significant reduction of alcohol consumption, recovery is expected in Mild-NCD-OH at both cerebral and cognitive levels, as opposed to the progressive degeneration provoking the transition from Mild- to Major-NCD-AD.^46^ However, without follow-up of the patient or if excessive alcohol consumption is maintained, severity of either MTL or episodic memory damage does not appear as a reliable marker of Alzheimer's Disease when compared to OH. Present results in Mild-NCD patients showed that Mild-NCD-AD patients had lower metabolism than Mild-NCD-OH patients in the posterior precuneus. The presence of hypometabolism in the precuneus is also well-known as an early marker of Alzheimer's Disease pathology.^34,47,48^ However, Mild-NCD-OH patients had lower metabolism than Mild-NCD-AD patients in the anterior part of the precuneus, and the whole region was commonly altered in both groups when comparing to healthy controls. Hypometabolism in the precuneus is expected in Mild-NCD-OH patients,^33^ and this variable does not appear as a reliable marker of Alzheimer's Disease at the individual level. These different elements facilitating differential diagnosis in patients with Mild-NCD remain crucial, as this stage represents a key window of opportunity for providing the most appropriate care to patients in order to slow or prevent progression to Major-NCD.

This is even more challenging for more severely impaired patients, since no differences based on posteromedial hypometabolism (including the precuneus), medial temporal lobe damage (both functional and structural), or memory performance have been found between Major-NCD patients. Similar episodic memory deficits between Major-NCD-AD and Major-NCD-OH patients (i.e. patients with Korsakoff’s Syndrome) reflect amnesia in both pathologies, accordingly with inclusion criteria and consistently with the literature.^10,11^ Differential diagnosis between Major-NCD-AD and Major-NCD-OH patients has often been considered as straightforward, primarily based on patients’ clinical history (i.e. alcohol abuse, Wernicke Encephalopathy, …) and some symptoms considered pathognomonic of Major-NCD-OH (i.e. fantastic confabulations). However, accessing a reliable medical history is not always possible, especially in amnesic patients. When it is available, a history of Wernicke Encephalopathy is not systematically documented^49^ in Major-NCD-OH patient and is a risk factor of Major-NCD-AD.^5^ Moreover, while fantastic confabulations are considered characteristic of Major-NCD-OH, they are more generally associated with amnesia and do not allow differentiation between Major-NCD-AD and Major-NCD-OH patients.^50^ Still, previous studies have shown that Major-NCD patients might differ qualitatively in the specificity of the alteration in memory processes. For instance, episodic deficits are associated with an inability to retrieve information for Major-NCD-OH patients while the same deficits are better explained by a deficit in information storage in Major-NCD-AD patients.^12^ A thorough memory assessment may help differentiate Major-NCD-AD from Major-NCD-OH patients from a neuropsychological point of view.^11^ Herein and consistently with the literature, Major-NCD-AD patients had worse deficits than Major-NCD-OH patients in executive functioning and processing speed.^10^ We found visuo-construction and working memory deficits in Major-NCD-AD only, while such impairments have been previously reported in Major-NCD-OH patients also.^9^ Both pathologies can present heterogenous neuropsychological profiles, notably in Major-NCD-OH where executive deficits are not systematic.^9^ Once again, performance changes with follow-up can guide the diagnosis since no further degradation is expected in abstinent Major-NCD-OH patients,^51^ unlike in Alzheimer's Disease. Lack of strictly AD-specific alteration underlines how alcohol damage could mask Alzheimer's Disease development in case of comorbidity. Repeated assessment, both cognitive and cerebral, therefore appears crucial. Repeated neuropsychological assessment may potentially be more accessible in clinical settings, and our results suggest its relevance, particularly in amnesic patients.

This study has some limitations. Its cross-sectional design restrains the inferences possible regarding the evolution of NCD. In addition, we are fully aware that evolution from Mild- to Major-NCD differs across aetiologies, with Mild-NCD-AD being considered as prodromal of Major-NCD-AD, which does not parallel the two distinct clinical forms in NCD-OH. However, for both groups of Major-NCD, patients used to belong to the Mild-NCD group of their respective pathology, since only KS patients with a history of alcohol use disorders where included. Future studies using longitudinal design would allow more precision regarding the evolution across severity stages. Sample size was also relatively small across groups but we must highlight the rarity of precisely and well-defined groups of patients with multimodal data, notably for the Major-NCD-OH groups. No amyloid measure was available for NCD-OH patients and no precise alcohol consumption measures were available for NCD-AD patients. Additional medical data in all the groups included (e.g. cardiovascular health or smoking) would have enabled us to better adjust for confounding factors. However, the originality and reliability of our study remain intact, notably because the absence of biomarkers is counterbalanced by the strict inclusion and exclusion criteria of the protocols, as well as the longitudinal follow-up, which helped rule out comorbidities in our cohort. Our work is a starting point of comparative research between NCD-OH and NCD-AD and paves the way to future studies that need to investigate Alzheimer's Disease biomarkers in NCD-OH patients and assess possible interactive effect of alcohol and Alzheimer's Disease pathology.^6^ For example, describing the neurocognitive profiles of patients with a dual diagnosis or Alzheimer's Disease patients with a history of alcohol-related pathologies would be particularly relevant. Even though it is challenging to recruit older patients with NCD-OH and free of comorbidities, future prospective studies should also compare patients who are matched on demographic variables, notably age, whose effect needs to be both controlled for and compared across aetiologies. Sex effects were not investigated but only controlled for, due to a high male frequency in Mild-NCD-OH patients, which matches the population encountered in Addiction departments. Other cognitive functions, not studied herein, could be specifically affected in NCD-OH patients. For instance, we could expect that psychomotor^45^ abilities are specifically altered in NCD-OH patients, notably in line with cerebellar alterations.

Beyond these methodological considerations, our findings raise important implications for the care of patients with NCD-OH and/or NCD-AD. The striking overlaps in neurocognitive alterations between NCD-OH and NCD-AD phenotypes underline how difficult it can be to identify a dual diagnosis. Misdiagnosis or delayed diagnosis can distort prognostic predictions and risk assessment. This can be detrimental to clinician decision-making (e.g. prescription of medication, referral to other services and cognitive remediation). Further research is needed to develop specific biomarkers and provide diagnostic tools compatible with clinical reality. In the meantime, it seems necessary to promote longitudinal follow-up of patients, with assessments including but not limited to a precise neuropsychological evaluation and brain imaging. Clinical interviews should be thorough and include key anamnestic elements, particularly with regard to alcohol consumption history, which can also be objectified on the basis of consumption markers such as phosphatidylethanol.

## Conclusion

Our study provides indications to help clinicians in identifying the aetiology of symptoms in patients who could have NCD due to Alzheimer's Disease, alcohol, or both, and thus contributes to the improvement of patient care. The specificities we underlined were characteristic of the pathologies by the severity of impairments but most of these cognitive functions or cerebral structures were affected in both aetiologies, except for cerebellar abnormalities that appeared specific to alcohol. The striking overlaps in alterations between NCD-OH and NCD-AD phenotypes make it difficult to distinguish between these phenotypes, or identifying dual diagnoses, based on neurocognitive symptoms alone. It highlights the importance of questioning Alzheimer's Disease in NCD-OH patients and vice versa. Impairments common to both diseases highlight the involvement of the Papez Circuit.

## Supplementary Material

fcaf289_Supplementary_Data

## Data Availability

De-identified data supporting the findings of the study can be made available upon request from the corresponding author: anne-lise.pitel@unicaen.fr.
