# Supplementary material for: Cognitive and cerebral phenotypes of neurocognitive disorders due to alcohol or Alzheimer’s disease
Source: Brain Commun. 2025 Aug 20;7(4):fcaf289. doi: 10.1093/braincomms/fcaf289 (PMC12366719; doi:10.1093/braincomms/fcaf289)
Supplement: fcaf289_Supplementary_Data [file fcaf289_supplementary_data.docx]

**Table of content**

[Supplementary Table 1 – detailed statistics for every factor included in ANCOVAs on cognitive variables 2](#_Toc199763556)

[Supplementary Table 2 – detailed statistics for every factor included in ANCOVAS on extracted values from significant clusters (imaging modalities) 3](#_Toc199763557)

[Supplementary Method - Determination of Amyloid status 4](#_Toc199763558)

Supplementary [Figure 1 - Neuroimaging profiles of mild neurocognitive disorders patients when compared to controls. 5](#_Toc199763559)

Supplementary [Figure 2 - Neuroimaging profiles of major neurocognitive disorders patients when compared to controls. 6](#_Toc199763560)

[References 7](#_Toc199763561)

|  | **Group** | **Age** | **Sex** | **Education** |
| --- | --- | --- | --- | --- |
| ***Episodic memory*** | *F*(4,161)=77.58; *p*<0.001; ***η2*=0.70** | *F*(1,161)=11.32; *p*<0.001; ***η2*=0.01** | *F*(1,161)=7.67; *p*=0.006; ***η2*=0.01** | *F*(1,161)=16.05; *p*<0.001; ***η2*=0.02** |
| ***Executive functions*** | *F*(4,195)=57.66; *p*<0.001; ***η2*=0.58** | *F*(1,195)=0.07; *p*=0.78; ***η2*<0.001** | *F*(1,195)=1.79; *p*=0.18; ***η2*=0.002** | *F*(1,195)=13.50; *p*<0.001; ***η2*=0.03** |
| ***Working memory*** | *F*(4,195)=4.35; *p*=0.002; ***η2*=0.18** | *F*(1,195)=3.90; *p*=0.050; ***η2*=0.02** | *F*(1,195)=0.00; *p*=0.980; ***η2*<0.001** | *F*(1,195)=19.06; *p*=<0.001; ***η2*=0.07** |
| ***Processing speed*** | *F*(4,195)=32.90; *p*<0.001; ***η2*=0.49** | *F*(1,195)=4,00; *p*<0.047; ***η2*=0.01** | *F*(1,195)=4.63; *p*=0.033; ***η2*=0.008** | *F*(1,195)=9.33; *p*=0.003; ***η2*=0.02** |
| ***Visuoconstruction*** | *F*(4,161)=23.93; *p*<0.001; ***η2*=0.38** | *F*(1,161)=1.66; *p*=0.198; ***η2*=0.01** | *F*(1,161)=3.16; *p*=0.077; ***η2*=0.01** | *F*(1,161)=15.58; *p*<0.001; ***η2*=0.05** |

# Supplementary Table 1 – detailed statistics for every factor included in ANCOVAs on cognitive variables

This table detailed statistics of each term included in ANCOVA models for cognition. Eta2 are in bold, to highlight that variance is always mainly explained by the group and only moderately by demographics.

# Supplementary Table 2 – detailed statistics for every factor included in ANCOVAS on extracted values from significant clusters (imaging modalities)

| **Imaging modality** | **Contrasts** | **Groupe** | **AGE** | **Sex** | **Education** | **TIV** |
| --- | --- | --- | --- | --- | --- | --- |
| ***Grey matter* (total volume)** | ***Conjunction Mild-NCD*** | *F*(4,193)=24; *p*<0.001; **η2=0.4** | *F*(1,193)=64; *p*<0.001; **η2=0.1** | *F*(1,193)=0.5; *p*=0.5; **η2=0.1** | *F*(1,193)=0.4; *p*=0.6; **η2 <0.001** | *F*(1,193)=88; *p*<0.001; **η2=0.1** |
|  | ***Mild-NCD-OH < Mild-NCD-AD*** | *F*(4,193)=35; *p*<0.001; **η2=0.3** | *F*(1,193)=244; *p*<0.001; **η2=0.3** | *F*(1,193)=4; *p*=0.04; **η2=0.1** | *F*(1,193)=1; *p*=0.3; **η2=0.004** | *F*(1,193)=80; *p*<0.001; **η2=0.08** |
|  | ***Mild-NCD-AD < Mild-NCD-OH*** | *F*(4,193)=15; *p*<0.001; **η2=0.3** | *F*(1,193)=26; *p*<0.001; **η2=0.06** | *F*(1,193)=0.04; *p*=0.8; **η2=0.1** | *F*(1,193)=2; *p*=0.2; **η2 <0.001** | *F*(1,193)=120; *p*<0.001; **η2=0.2** |
|  | ***Conjunction Major-NCD*** | *F*(4,193)=33; *p*<0.001; ***η2*=0.5** | *F*(1,193)=147; *p*<0.001; ***η2*=0.2** | *F*(1,193)=0.01; *p*=0.9; ***η2*=0.09** | *F*(1,193)=1; *p*=0.3; ***η2*=0.004** | *F*(1,193)=106; *p*<0.001; ***η2*=0.1** |
|  | ***Major-NCD-OH < Major-NCD-AD*** | *F*(4,193)=28; *p*<0.001; ***η2*=0.3** | *F*(1,193)=144; *p*<0.001; ***η2*=0.2** | *F*(1,193)=3; *p*=0.08; ***η2*=0.1** | *F*(1,193)=0.2; *p*=0.7; ***η2*=0.002** | *F*(1,193)=80; *p*<0.001; ***η2*=0.1** |
| ***White matter* (total volume)** | ***Mild-NCD-OH < Mild-NCD-AD*** | *F*(4,193)=14; *p*<0.001; ***η2*=0.2** | *F*(1,193)=5; *p*=0.03; ***η2*=0.02** | *F*(1,193)=0.04; *p*=0.8; ***η2*=0.2** | *F*(1,193)=4; *p*=0.06; ***η2* <0.001** | *F*(1,193)=159; *p*<0.001; ***η2*=0.2** |
|  | ***Mild-NCD-AD < Mild-NCD-OH*** | *F*(4,193)=11; *p*<0.001; ***η2*=0.1** | *F*(1,193)=12; *p*<0.001; ***η2*=0.02** | *F*(1,193)=0.3; *p*=0.6; ***η2*=0.1** | *F*(1,193)=2; *p*=0.2; ***η2*=0.001** | *F*(1,193)=46; *p*<0.001; ***η2*=0.1** |
|  | ***Conjunction Major-NCD*** | *F*(4,193)=14; *p*<0.001; ***η2*=0.3** | *F*(1,193)=4; *p*=0.04; ***η2*=0.02** | *F*(1,193)=0.03; *p*=0.9; ***η2*=0.2** | *F*(1,193)=4; *p*=0.05; ***η2* <0.001** | *F*(1,193)=144; *p*<0.001; ***η2*=0.2** |
|  | ***Major-NCD-OH < Mild-NCD-AD*** | *F*(4,193)=13; *p*<0.001; ***η2*=0.2** | *F*(1,193)=17; *p*<0.001; ***η2*=0.04** | *F*(1,193)=0.07; *p*=0.8; ***η2*=0.2** | *F*(1,193)=5; *p*=0.03; ***η2*=0.001** | *F*(1,193)=175; *p*<0.001; ***η2*=0.2** |
| ***Metabolism* (mean SUVRr glucose value)** | ***Conjunction Mild-NCD*** | *F*(4,184)=44; *p*<0.001; ***η2*=0.6** | *F*(1,184)=59; *p*<0.001; ***η2*=0.09** | *F*(1,184)=17; *p*<0.001; ***η2*=0.03** | *F*(1,184)=0.07; *p*=0.8; ***η2* <0.001** | NA |
|  | ***Mild-NCD-OH < Mild-NCD-AD*** | *F*(4,184)=26; *p*<0.001; ***η2*=0.3** | *F*(1,184)=94; *p*<0.001; ***η2*=0.2** | *F*(1,184)=0.3; *p*=0.6; ***η2* <0.001** | *F*(1,184)=0.1; *p*=0.7; ***η2* <0.001** | NA |
|  | ***Mild-NCD-AD < Mild-NCD-OH*** | *F*(4,184)=32; *p*<0.001; ***η2*=0.5** | *F*(1,184)=0.5; *p*=0.5; ***η2* <0.001** | *F*(1,184)=8; *p*=0.006; ***η2*=0.02** | *F*(1,184)=0.03; *p*=0.9; ***η2* <0.001** | NA |
|  | ***Conjunction Major-NCD*** | *F*(4,184)=28; *p*<0.001; ***η2*=0.5** | *F*(1,184)=70; *p*<0.001; ***η2*=0.1** | *F*(1,184)=10; *p*=0.002; ***η2*=0.02** | *F*(1,184)=0.6; *p*=0.4; ***η2*=0.001** | NA |
|  | ***Major-NCD-AD < Major-NCD-OH*** | *F*(4,184)=27; *p*<0.001; ***η2*=0.5** | *F*(1,184)=8; *p*=0.004; ***η2*=0.02** | *F*(1,184)=7; *p*=0.008; ***η2*=0.02** | *F*(1,184)=0.4; *p*=0.5; ***η2* <0.001** | NA |
|  | ***Major-NCD-OH < Major-NCD-AD*** | *F*(4,184)=36; *p*<0.001; ***η2*=0.4** | *F*(1,184)=135; *p*<0.001; ***η2*=0.2** | *F*(1,184)=12; *p*<0.001; ***η2*=0.02** | *F*(1,184)=0.09; *p*=0.8; ***η2* <0.001** | NA |

For each modality and contrast, we extracted for all patients and participants the values of the significant clusters (i.e., total volume / mean glucose SUVr value), then applied an ANCOVA model correcting for age, sex, education (and TIV for structural measures). This table detailed statistics of each term included in ANCOVA models for cognition. Eta2 are in bold, to highlight that variance is always mainly explained by the group and only moderately by demographics.

# Supplementary Method

## Determination of Amyloid status

18F-AV45 PET (Fluorine-18 florbetapir positron emission tomography) images were co-registered onto their corresponding MRI and normalized to the MNI template using deformation parameters from the T1-weighted normalization procedure. Images were then quantitatively normalized using the cerebellar grey matter as the reference region, resulting in standardized uptake value ratios (SUVRs). Amyloid uptake was extracted and averaged across AD-sensitive regions, including the frontal, temporal and parietal cortices, the precuneus, the anterior striatum, and the insular cortex.^1^ SUVRs were transformed to Centiloid values by applying the standardized procedure proposed by Klunk and collaborators.^2^

For validation steps, we first assessed the zero-to-100 Centiloid scale from the same data as them (PiB-PET data from GAAIN) and confirmed that our results matched theirs, demonstrating our ability to reproduce their findings. We also preprocessed their data with our pipeline to assess any variations in results. Our in-home preprocessing yielded similar outcomes, meeting validation criteria. Given that Klunk and collaborators’ procedure was designed for the PiB tracer, while our study employed Florbetapir, we applied the conversion procedure proposed by Navitsky and collaborators using their data, which encompassed both PiB- and Florbetapir-PET imaging in the same individuals ^3^. We derived the zero-to-100 Centiloid scale in PiB-PET data and then applied the conversion formula to translate Florbetapir SUVRs from PET volumes of interest into the Centiloid scale. The correlation between PiB- and Florbetapir-derived Centiloid values satisfied validation criteria. Using this conversion procedure, we processed our Florbetapir-PET scans and used a cutoff of 12 Centiloid, based on previous work,^4^ to define a positive amyloid status.

## R Code

# -------------------------------------------------------------------

# Title: R script for ANCOVA and post-hoc analyses on neuropsychological variables

# Corresponding manuscript: COGNITIVE AND CEREBRAL PHENOTYPES OF NEUROCOGNITIVE DISORDERS DUE TO ALCOHOL OR ALZHEIMER'S DISEASE

# Author: Célia Soussi

# -------------------------------------------------------------------

# --- Load required packages ---

library(car) # For type III ANOVA

library(multcomp) # For Tukey post-hoc tests

library(effectsize) # For computing partial eta squared

# --- Initialize results list ---

results <- list()

# --- Loop through each neuropsychological variable ---

# This loop computes an ANCOVA for each variable listed in var_neuropsy,

# using Group as the main predictor and Age, Sex, and Education as covariates.

# The data frame 'data' must contain all relevant variables.

for (var in var_neuropsy) {

message("Processing variable: ", var)

model <- lm(data[[var]] ~ Group + Age + Sex + Education, data = data)

anova_result <- Anova(model, type = 3)

print(anova_result)

p_value <- anova_result["Group", "Pr(>F)"]

eta2 <- eta_squared(anova_result, partial = TRUE)

results[[var]] <- list(

anova = anova_result,

eta_squared = eta2,

p_value = p_value

)

# --- Post-hoc analysis (Tukey) if Group effect is significant ---

if (!is.na(p_value) && p_value < 0.05) {

tukey_test <- glht(model, linfct = mcp(Group = "Tukey"))

tukey_summary <- summary(tukey_test)

tukey_ci <- confint(tukey_test)

results[[var]]$tukey <- tukey_summary

results[[var]]$confint <- tukey_ci

print(tukey_summary)

print(tukey_ci)

}

}

# --- Access results for a specific variable ---

# Example: results[["VariableName"]]$anova

# $tukey

# $eta_squared

# Supplementary Figure 1 - Neuroimaging profiles of mild neurocognitive disorders patients when compared to controls.


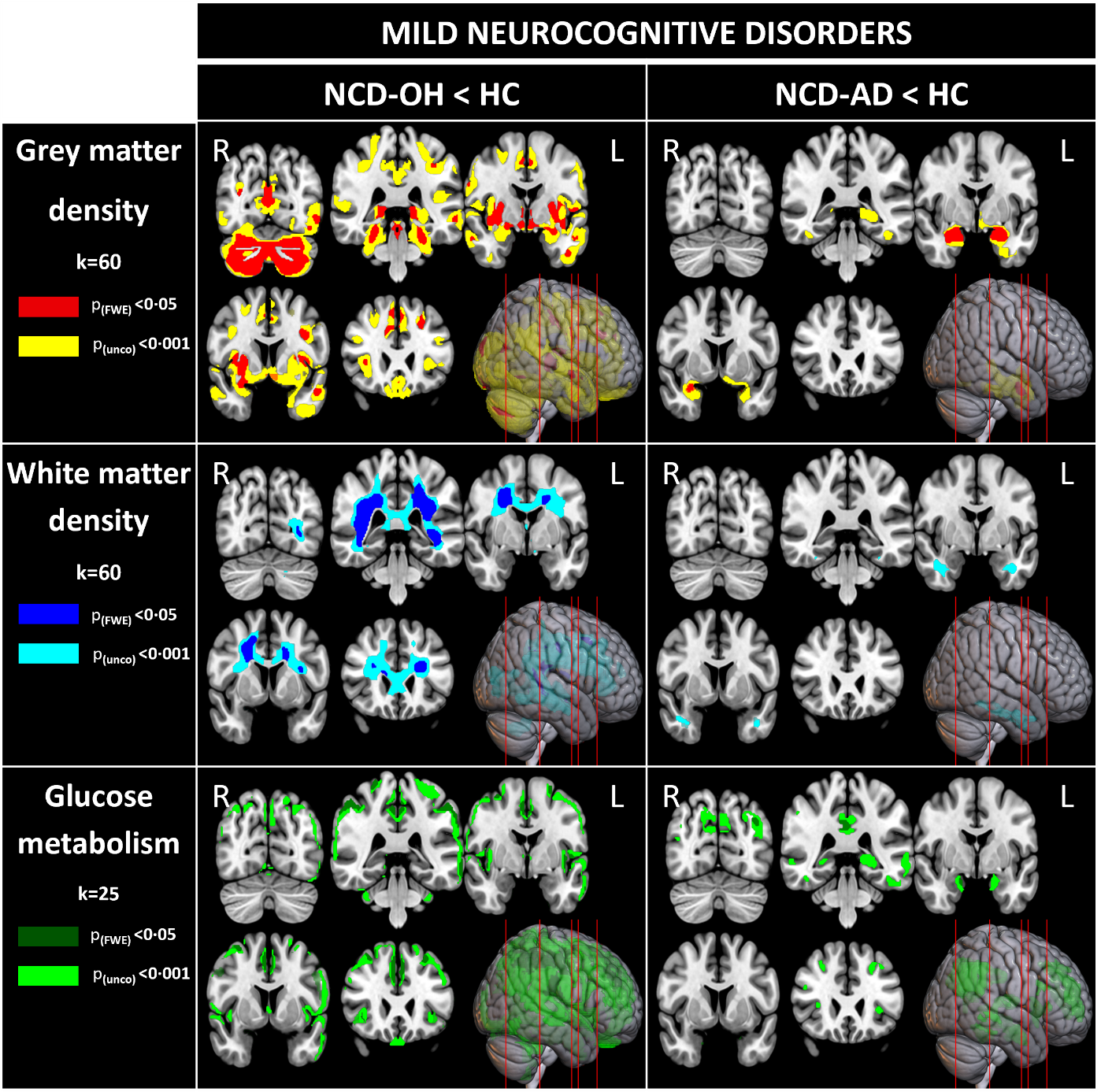


**Neuroimaging profiles of mild neurocognitive disorders patients when compared to controls.**

Voxel-wised analyses were carried out using a full factorial model with the group as a factor and total intracranial volume (TIV), sex, age, and education as covariates. Same analyses were run without TIV as covariate for PET modality. Significant results for each investigated contrast have been projected on the same five axonal sections and rendered on a lateral view of the brain. Each column corresponds to a comparison between the patient group and control participants (HC, *n*=81), with a patient < HC contrast (*left:* Mild-NCD-OH patients (n=50), *right:* Mild-NCD-AD patients (*n*=30)) Each row corresponds to an imaging modality (*from top to bottom*: grey matter density, white matter density, glucose metabolism). For each imaging modality, legend in the left column indicates color codes for each level of correction presented (either corrected for Family-Wise-Error (FEW) at *p*<0.05 or uncorrected for multiple comparison at *p*<0.001. For the glucose metabolism modality, the sample size for the Mild-NCD-OH group was n = 42.

# Supplementary Figure 2 - Neuroimaging profiles of major neurocognitive disorders patients when compared to controls.


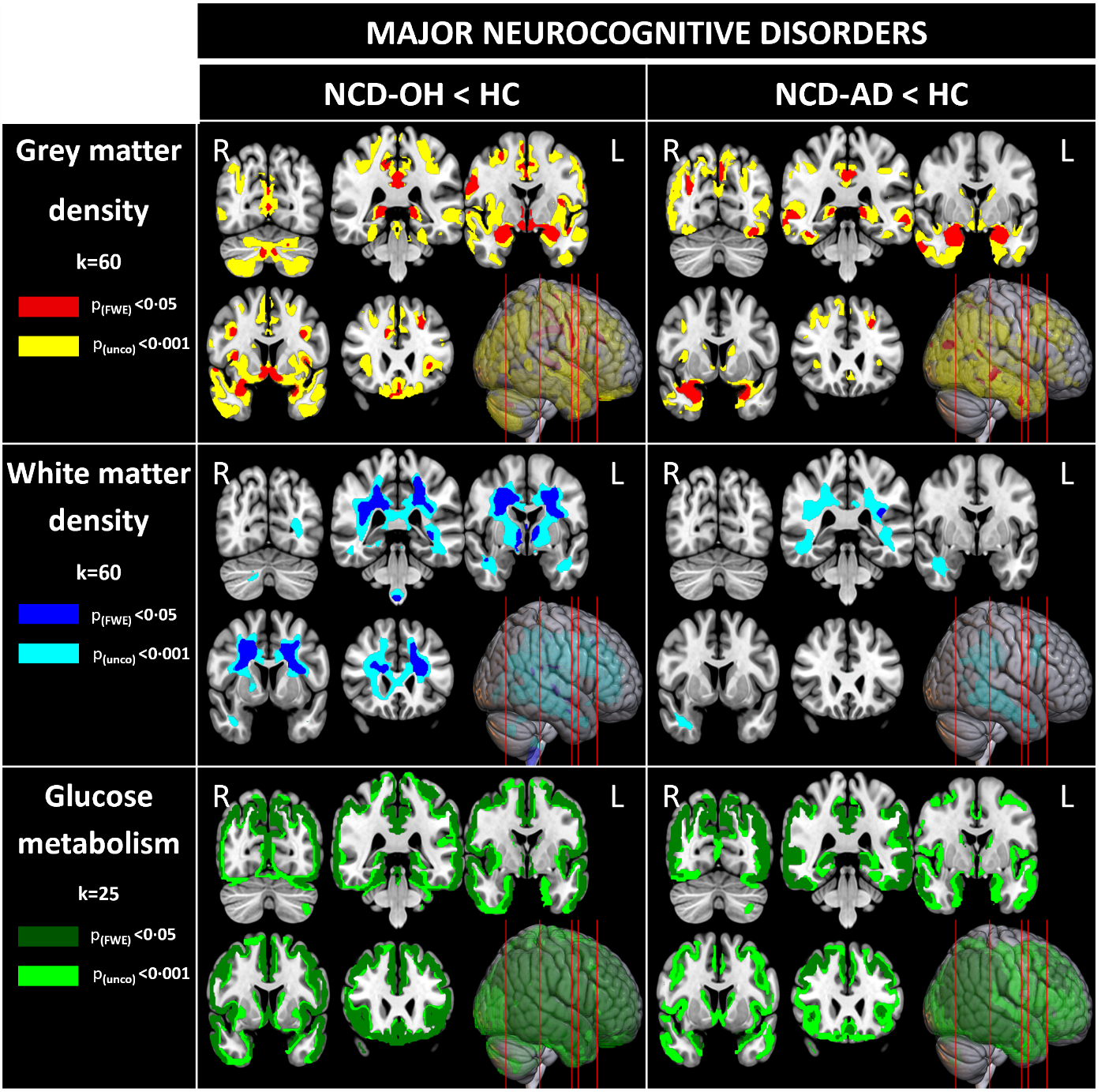


**Neuroimaging profiles of major neurocognitive disorders patients when compared to controls.**

Voxel-wised analyses were carried out using a full factorial model with the group as a factor and total intracranial volume (TIV), sex, age, and education as covariates. Same analyses were run without TIV as covariate for PET modality. Significant results for each investigated contrast have been projected on the same five axonal sections and rendered on a lateral view of the brain. Each column corresponds to a comparison between the patient group and control participants (HC *n*=81), with a patient < HC contrast (left: Major-NCD-OH patients (*n*=18), right: Major-NCD-AD patients (*n*=24)) Each row corresponds to an imaging modality (from top to bottom: grey matter density, white matter density, glucose metabolism). For each imaging modality, legend in the left column indicates color codes for each level of correction presented (either corrected for Family-Wise-Error (FEW) at *p*<0.05 or uncorrected for multiple comparison at *p*<0.001). For the glucose metabolism modality, the sample size for the Major-NCD-AD group was n = 23.

# References

1. La Joie R, Perrotin A, de La Sayette V, et al. Hippocampal subfield volumetry in mild cognitive impairment, Alzheimer’s disease and semantic dementia. *NeuroImage: Clinical*. 2013;3:155-162. doi:10.1016/j.nicl.2013.08.007

2. Klunk WE, Koeppe RA, Price JC, et al. The Centiloid Project: standardizing quantitative amyloid plaque estimation by PET. *Alzheimer’s &amp; dementia : the journal of the Alzheimer’s Association*. 2015;11(1):1—15.e1—4. doi:10.1016/j.jalz.2014.07.003

3. Navitsky M, Joshi AD, Kennedy I, et al. Standardization of amyloid quantitation with florbetapir standardized uptake value ratios to the Centiloid scale. *Alzheimer’s & Dementia*. 2018;14(12):1565-1571. doi:10.1016/j.jalz.2018.06.1353

4. La Joie R, Ayakta N, Seeley WW, et al. Multisite study of the relationships between *antemortem* [11C]PIB-PET Centiloid values and *postmortem* measures of Alzheimer’s disease neuropathology. *Alzheimer’s & Dementia*. 2019;15(2):205-216. doi:10.1016/j.jalz.2018.09.001
